# Freeze–Thaw-Synthesized PVA/Chitosan Hydrogels: Structure–Property Relationships and ANN Modeling of Swelling and Degradation Behaviors

**DOI:** 10.1021/acsomega.6c00485

**Published:** 2026-04-08

**Authors:** Elif İlayda Tarım, Cihangir Boztepe, Mahmut Daskin, Gozde Ozaydin İnce

**Affiliations:** † Department of Biomedical Engineering, Faculty of Engineering, 37520 Inonu University, 44280 Malatya, Turkiye; ‡ Department of Mechanical Engineering, Faculty of Engineering, Inonu University, 44280 Malatya, Turkiye; § Department of Material Science and Nanoengineering, Faculty of Engineering and Natural Sciences, Sabanci University, 34956 Istanbul, Turkiye

## Abstract

Reliable prediction of swelling and degradation behavior of hydrogels used in biomedical applications due to the simultaneous and nonlinear effects of multiple parameters is a critical requirement in hydrogel design. However, conventional experimental approaches present significant limitations in multiparameter systems due to high time consumption and experimental costs. In this study, the structural, swelling, and degradation characteristics of poly­(vinyl alcohol)/chitosan (PVA/CS) composite hydrogels synthesized using a physical cross-linking method based on freeze–thaw (F–T) cycles with varying chitosan contents and F–T cycle numbers were systematically investigated. To prioritize biocompatibility, the hydrogels were produced without the use of chemical cross-linking agents, and the effects of pH-sensitive behavior, network density, and structural stability were examined in detail. FT-IR analyses confirmed the formation of hydrogen bonding interactions and an IPN-like network structure between PVA and chitosan chains, while SEM observations revealed significant changes in pore morphology depending on chitosan content and the number of F–T cycles. The swelling capacities of the hydrogels were found to vary between 4.02 and 26.28 g water/g polymer, whereas degradation ratios ranged from 3.38% to 37.36%, depending on the environmental pH, chitosan concentration, and F–T cycle number. The experimentally obtained nonlinear swelling and degradation data were further modeled using an artificial neural network (ANN) approach, and the behaviors were predicted with high accuracy (*R* > 0.99). The results demonstrate that ANN-based modeling provides a reliable and efficient design and optimization tool for multiparameter hydrogel systems by significantly reducing experimental workload.

## Graphical abstract

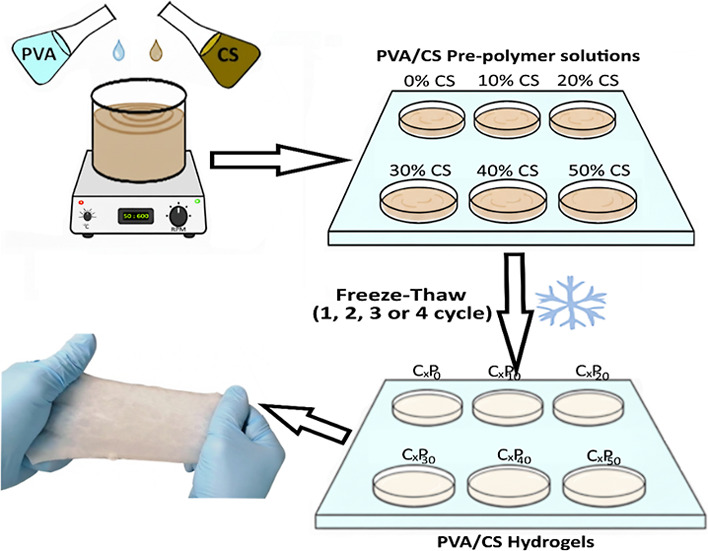

## Introduction

Hydrogels are important biomedical materials that exhibit mechanical and physicochemical properties comparable to those of soft tissues due to their high water-holding capacity and three-dimensional polymer network structures. Their high water content (70–99%) enables hydrogels to create a microenvironment compatible with biological tissues, providing a significant advantage, particularly in applications involving direct contact with biological environments.^1,2^ Hydrophilic functional groups within their structure (such as –OH, –NH_2_, and –COOH) interact with water molecules through hydrogen bonding, facilitating swelling behavior and contributing to the maintenance of the moist microenvironment required for cell viability. These characteristics make hydrogels among the most preferred materials in terms of biocompatibility.^3,4^

In the biomedical field, hydrogels are widely used in various applications, including controlled drug delivery, wound healing, and tissue engineering. Owing to their tunable polymer network structures, hydrogels enable controlled and targeted release of therapeutic agents while simultaneously promoting cell migration and tissue regeneration in wound healing applications by maintaining a moist environment. In tissue engineering, hydrogels play a critical role in regenerative medicine by providing suitable three-dimensional scaffold structures that support cell adhesion and proliferation.^5,6^

Poly­(vinyl alcohol) (PVA) is a synthetic polymer widely used in biomedical hydrogel systems due to its high biocompatibility, mechanical stability, and ease of processability. In particular, the ability of PVA to be converted into hydrogels via physical cross-linking methods, such as freeze–thaw (F–T) cycling, without the need for chemical cross-linking agents, renders PVA-based hydrogels safe and attractive for biomedical applications.^7,8^ Chitosan, on the other hand, is a naturally derived, biodegradable, and biocompatible polysaccharide that has attracted considerable attention due to its intrinsic antibacterial properties and pH-responsive behavior. Owing to the presence of amino groups in its molecular structure, chitosan can respond to environmental pH variations, enabling effective control over the swelling and degradation behavior of hydrogel systems.^9,10^

Composite hydrogels formed by combining PVA and chitosan exhibit a synergistic structure that integrates the mechanical strength of PVA with the biological functionality and pH responsiveness of chitosan. This synergistic interaction enables precise tuning of the hydrogel’s swelling, degradation, and mechanical properties, thereby providing a versatile and adaptable material platform for biomedical applications.^11,12^

Freeze–thaw (F–T) cycling is an effective physical cross-linking approach that enhances the biocompatibility of hydrogels by eliminating the need for chemical cross-linking agents. The number of freeze–thaw cycles directly influences the density and pore morphology of the polymer network; increasing the number of cycles leads to a more compact network structure, reduced pore size, and enhanced mechanical strength. Therefore, precise control of freeze–thaw parameters is critical for tailoring hydrogel properties to meet the requirements of specific biomedical applications.^13,14^

However, the swelling and degradation behavior of hydrogel systems is governed by the simultaneous influence of multiple parameters, including pH, polymer composition, chitosan content, and cross-linking density. The nonlinear interactions among these parameters hinder comprehensive evaluation using purely experimental approaches, thereby necessitating a large number of experiments. This increased experimental demand, in turn, imposes significant constraints in terms of time and cost.^15,16^

At this stage, artificial neural networks (ANNs) offer a powerful alternative for modeling multi-input, nonlinear systems. Previous studies have reported that ANN-based models achieve high accuracy in predicting swelling and degradation behavior in hydrogel systems. However, studies that integrate experimental investigations with ANN-based modeling while simultaneously evaluating key parameters such as pH, chitosan content, and freeze–thaw cycle number remain limited.^17,18^

The originality of this study lies not only in the experimental investigation of the pH-sensitive swelling and degradation behavior of PVA/chitosan-based hydrogels but also in the simultaneous and holistic prediction of these behaviors using a multiparameter ANN model. In contrast to most existing studies,^19–24^ which typically assess hydrogel behavior based on single parameters or limited variable combinations and present the results in a largely descriptive manner, this work incorporates fundamental parameters directly influencing the hydrogel network structure—namely, pH, chitosan content, and freeze–thaw cycle number—within a unified modeling framework. A framework that simultaneously parametrizes (pH, chitosan content, FT cycle number) as structural determinants and links them quantitatively to network descriptors (e.g., porosity, swelling, and degradation) would therefore go beyond the predominantly descriptive, factor-by-factor approaches evident in current chitosan-hydrogel literature. By employing an ANN approach capable of learning complex nonlinear interactions with high accuracy, this holistic strategy enables reliable prediction of hydrogel behavior, reduces the experimental burden, and accelerates the hydrogel design process. Consequently, the present study contributes to the literature by introducing an original, predictive, and decision-support methodology that complements experimental investigations in the design and optimization of multiparameter hydrogel systems.

## Materials and Methods

### Materials

Poly­(vinyl alcohol) (PVA, molecular weight 98,000 and 99+% hydrolyzed) and acetic acid were purchased from Sigma-Aldrich (Germany). Chitosan (CS, molecular weight 190,000–375,000 and 75–85% deacetylated) was purchased from ABCR (Germany). All chemicals were used as received without any further purification, and deionized water was used in all of the experiments.

### Preparation of PVA/Chitosan Hydrogels

PVA/chitosan hydrogels were synthesized by using the freeze–thaw (F–T) method. Initially, a 15 wt % aqueous PVA solution was prepared by dissolving PVA powder in distilled water at 85 °C under magnetic stirring for 3 h until complete dissolution, resulting in a homogeneous and viscous PVA solution. Separately, a chitosan solution containing 3 wt % chitosan was prepared by dissolving chitosan in a 2% (v/v) acetic acid aqueous solution. Using the prepared PVA and chitosan solutions, six different PVA/chitosan mixtures containing 0, 10, 20, 30, 40, and 50 vol % chitosan solution were obtained. These mixtures were magnetically stirred at 40 °C for 3 h to ensure homogeneity. Subsequently, 25 mL of each mixture was poured into a glass Petri dish with a diameter of 10 cm. To obtain hydrogels with different cross-linking densities, each PVA/chitosan solution was subjected to 1, 2, 3, and 4 freeze–thaw cycles, resulting in a total of 24 distinct hydrogel formulations based on the composition and cross-linking degree. One freeze–thaw cycle consisted of freezing the PVA/chitosan solution at −20 °C for 18 h, followed by thawing at room temperature for 3 h. The synthesized hydrogels were systematically coded according to their synthesis parameters and the pH conditions used during testing. The coding system included the number of freeze–thaw cycles, the volumetric percentage of the chitosan solution in the hydrogel formulation, and the pH value of the test medium. The hydrogels were denoted as C*x*P*y*pH*z*, where *x* represents the number of freeze–thaw cycles, *y* indicates the volumetric percentage of the chitosan solution, and *z* corresponds to the pH value. For example, the code C_2_P_30_pH_9_ refers to a hydrogel synthesized with two freeze–thaw cycles, containing a 30 vol % chitosan solution, and tested at pH 9.

### Swelling Tests

The swelling behavior of PVA/chitosan hydrogels was investigated in phosphate-buffered saline (PBS) solutions with different pH values. For this purpose, the 24 synthesized hydrogel samples were cut into pieces with dimensions of 2 cm × 2 cm. The hydrogel specimens were first allowed to swell in distilled water for 3 days to reach equilibrium swelling. Subsequently, the equilibrium-swollen hydrogels were completely dried by lyophilization. After drying, the dry weight of each hydrogel sample was recorded, and the samples were then immersed in 1 wt % PBS solutions adjusted to pH values of 2, 5, 7, 9, and 12. The pH of the PBS solutions was adjusted by using HCl and NaOH solutions. A total of 120 swelling kinetic experiments were conducted, corresponding to 24 hydrogel formulations evaluated at five different pH levels. During the swelling experiments, the hydrogel samples were incubated in PBS solutions at 37 °C. The samples were periodically removed from the solutions at 24 h intervals over a period of 21 days, gently blotted to remove excess surface liquid, and weighed. The swelling (*S*) and equilibrium swelling (*S*_eq_) values of the PVA/chitosan hydrogels were calculated using eqs 1 and 2, respectively. In these equations, *S* represents the swelling degree of the hydrogel at time *t*, *S*_eq_ denotes the equilibrium water content (equilibrium swelling value), *m*_eq_ is the mass of the hydrogel at equilibrium in distilled water, and *m*_0_ is the dry mass of the hydrogel.

1 $$S_{eq}=m_{eq}-m_{0}/m_{0}$$

2 $$S=\frac{m_{t}-m_{0}}{m_{0}}$$

### Degradation Tests

Degradation studies were conducted in a manner similar to the swelling experiments using phosphate-buffered saline (PBS) solutions with different pH values. For this purpose, 24 lyophilized hydrogel samples were placed in capped vial tubes containing 1 wt % PBS solutions adjusted to pH values of 2, 5, 7, 9, and 12. The degradation experiments were carried out for 21 days in an incubator maintained at 37 °C. At predetermined time intervals of 24 h, the hydrogels were removed from the PBS solutions, dried, and weighed. The percentage degradation (% *D*) of each hydrogel sample was calculated based on the difference in dry mass before and after degradation, using eq 3. In this equation, % *D* represents the percentage degradation, and *m*_td_ denotes the dry mass of the hydrogel at time after incubation in the PBS solution.

3 $$D\;\left(\%\right)=\frac{m_{0}-m_{td}}{m_{0}}\times 100$$

All swelling and degradation experiments were performed in triplicate, and the results are reported as mean values.

### Morphological and Chemical Characterization

The surface morphology of the PVA/CS hydrogels was examined by using scanning electron microscopy (SEM, Leo-Evo 40XVP). The chemical composition and functional groups of the hydrogels were analyzed by Fourier transform infrared (FT-IR) spectroscopy using a PerkinElmer spectrometer (KBr pellet method) over the wavenumber range of 4000–400 cm^–1^.

### Mechanical Tests

The mechanical properties of the PVA/CS hydrogels were evaluated using an Instron 5943 Universal Testing System (300 LX, USA) fitted with a 100 N load cell. Experiments were performed at room temperature (22 °C) with a constant cross-head speed of 100 mm/min. Specifically, specimens measuring 15 × 60 × 1.5 mm were subjected to uniaxial tension until rupture to capture their characteristic stress–strain behavior. Engineering stress was calculated by dividing the tensile load by the initial cross-sectional area, while tensile strain was determined as the ratio of elongation to the original gauge length. The Young modulus for the PVA/CS hydrogels was derived from the gradient of the stress–strain curve in the initial linear regime (5–20% strain). Additionally, fracture toughness was determined by integrating the area under the stress–strain curve until the point of failure, whereas energy dissipation was calculated from the area enclosed within the loading–unloading loops.

### Artificial Neural Network (ANN) Modeling

Artificial neural network (ANN) modeling was employed to predict the swelling and degradation behaviors of the PVA/chitosan hydrogels. A three-layer feed-forward backpropagation neural network architecture was used for both swelling and degradation modeling. The ANN models were developed by using MATLAB software along with the Neural Network Toolbox. The Levenberg–Marquardt algorithm (trainlm) was selected as the training algorithm to enhance generalization performance and prevent overfitting by optimizing the network weights. Custom scripts written in the MATLAB environment were used to introduce the experimental data set into the network architecture and to perform the training process. During training, the optimal network configuration was determined based on performance metrics, and the finalized network structure was saved to construct the predictive model.

In swelling modeling, the input layer consisted of 4 parameters (F–T cycle, CS ratio, pH, and time), while the output layer predicted the swelling rate. These parameters were derived from the experimental design including 4 different F–T cycles (1, 2, 3, 4), 6 different chitosan ratios (0%, 10%, 20%, 30%, 40%, 50%), 5 different pH values (2, 4, 7, 10, 12), and 6 different time intervals (1, 2, 3, 4, and 5 days). This data set used for the ANN modeling of swelling behavior consists of a total of 720 unique data points. Each “data point” represents the average swelling ratio of a specific formulation under a specific pH condition at a specific time. The hidden layer comprised 20 neurons employing logarithmic sigmoid (logsig) activation functions, which enabled the effective learning of nonlinear relationships between the input and output variables. To evaluate the predictive capability and prevent overfitting, the data were partitioned into three subsets: 504 points for training (70%), 108 points for validation (15%), and 108 points for independent testing (15%).

For degradation modeling, a similar three-layer ANN architecture was adopted. The input layer was structured with 3 primary parameters: F–T cycle number, chitosan ratio, and pH value. These inputs represent the 120 unique experimental combinations (4 F–T cycles × 6 CS ratios × 5 pH levels) designed to evaluate the structural stability of the hydrogels. The output layer predicted the mass loss percentage. The hidden layer consisted of 10 neurons with logarithmic sigmoid (logarithmic) activation functions. To evaluate the predictive capability and prevent overfitting, the data were partitioned into three subsets: 84 points for training (70%), 18 points for validation (15%), and 18 points for independent testing (15%).

The predictive capabilities of the developed ANN models were assessed using previously unseen test data, and model performance was quantitatively evaluated using statistical metrics. Several statistical indicators, including the coefficient of determination (*R* ^2^), root-mean-square error (RMSE), mean square error (MSE), and mean absolute percentage error (MAPE), were employed to evaluate the accuracy and reliability of the constructed networks. The values of *R* ^2^, RMSE, MSE, and MAPE were calculated by using standard mathematical formulations, as presented in the following equations.

4 $$R^{2}={\left(\frac{\sum_{m=1}^{N}\left(x_{m}-\mathrm{x̅}\right)\left(y_{m}-\mathrm{y̅}\right)}{\sqrt{\sum_{m=1}^{N}{\left(x_{m}-\mathrm{x̅}\right)}^{2}}\sqrt{{\sum_{m=1}^{N}\left(y_{m}-\mathrm{y̅}\right)}^{2}}}\right)}^{2}$$

5 $$RMSE=\sqrt{\frac{\sum_{m=1}^{N}{\left(y_{m}-x_{m}\right)}^{2}}{N}}$$

6 $$MSE=\frac{1}{N}\sum_{m=1}^{N}{\left(y_{m}-x_{m}\right)}^{2}$$

7 $$MAPE\;\left(\%\right)=\frac{1}{N}\sum_{m=1}^{N}{\left(|\frac{y_{m}-x_{m}}{x_{m}}|\right)}^{2}\times 100$$

where *x*_m_ is an observed value at the *i*th time step, *y*_m_ is a simulated value at the same moment of time, *N* is the number of time steps, x̅ is the mean value of observations, and y̅ is the mean value of simulations in these equations.

## Results and Discussion

### Design Strategy and Structural Characterization of PVA/CS Hydrogels

The design strategy of PVA/chitosan (PVA/CS) composite hydrogels was established by considering biocompatibility, safety, and functional tunability, which are essential requirements for biomedical applications. In this context, the chitosan content and the number of freeze–thaw cycles were selected as the main parameters governing the structural and functional characteristics of the hydrogel systems and were systematically varied. Physical network structures were formed using a freeze–thaw approach without the incorporation of chemical cross-linking agents, thereby avoiding potential toxic residues and providing a safer hydrogel platform for biomedical use. Adjustment of the chitosan ratio introduced pH-responsive behavior and biological functionality to the hydrogels, whereas increasing the freeze–thaw cycle number enabled controlled modification of network density, pore morphology, and mechanical stability. The combined optimization of these parameters allowed the swelling and degradation behavior of the hydrogels to be tuned over a broad range, offering an integrated evaluation of material design for targeted biomedical applications. Table of contents (TOC) graphic schematically illustrates the experimental design strategy employed for the synthesis of PVA/chitosan (PVA/CS) composite hydrogels in this study. The scheme outlines the formation of physically cross-linked hydrogel networks obtained by subjecting PVA solutions containing different chitosan contents to various numbers of freeze–thaw cycles. This design approach highlights that simultaneous control of the chitosan ratio and freeze–thaw cycle number enables systematic tuning of the hydrogel network density, structural stability, and overall functional properties.

During freeze–thaw processing, PVA chains tend to move closer to each other within polymer-rich regions during the freezing step, leading to the formation of microcrystalline PVA domains. Upon thawing, these domains remain within the hydrogel matrix as permanent physical cross-linking sites. According to previous studies, such crystalline nodes are mainly responsible for the intrinsic mechanical stability of PVA-based hydrogels.^25,26^ An increase in the number of freeze–thaw cycles generally promotes the formation and effectiveness of these crystallites, resulting in higher network density, smaller pore dimensions, and enhanced mechanical stability.^27^ In PVA/chitosan (PVA/CS) composite hydrogels, the network architecture is further reinforced through hydrogen bonding and chain entanglements between PVA and chitosan chains. In particular, hydrogen bonds formed between the hydroxyl (–OH) groups of PVA and the amino (–NH_2_) and hydroxyl groups of chitosan contribute to the strengthening of physical cross-links, giving rise to a quasi-interpenetrating or IPN-like network structure.^28,29^ Increasing the chitosan content raises the density of hydrophilic functional groups, thereby making the pH-responsive behavior of the hydrogel more pronounced. Under acidic conditions, protonation of chitosan amino groups (–NH_3_^+^) enhances interchain electrostatic repulsion, which facilitates network expansion and accelerates swelling and degradation. In contrast, reduced protonation under basic conditions leads to a relatively more compact network structure. This pH-dependent mechanism is widely recognized as a key factor underlying the functional advantages of chitosan-based hydrogels in biomedical applications.^29,30^

The structural characterization of the synthesized PVA/chitosan (PVA/CS) hydrogel composites was carried out by using Fourier transform infrared (FT-IR) spectroscopy. The FT-IR spectra shown in Figure 1 were recorded in the range of 600–4000 cm^–1^ and evaluated based on the characteristic absorption bands corresponding to the functional groups present in the hydrogel structure. Figure 1A presents the FT-IR spectra of the neat PVA hydrogel without chitosan (i) and PVA/CS hydrogels containing 30% (ii) and 50% (iii) chitosan solution. In spectrum (i), the broad absorption band observed at 3313 cm^–1^ is attributed to O–H stretching vibrations originating from hydroxyl groups of PVA. The peaks located at 2936 and 2903 cm^–1^ correspond to aliphatic C–H stretching vibrations of methyl groups. The band observed at 1420 cm^–1^ is assigned to CH_2_ bending vibrations, while the sharp peak at 1094 cm^–1^ is associated with the symmetric stretching of aliphatic C–O–C groups in the PVA backbone. In addition, the peak around 848 cm^–1^ is related to out-of-plane C–H bending vibrations, which is consistent with previous reports.^31^ Upon incorporation of chitosan into the PVA matrix, noticeable changes in the FT-IR spectra are observed. The O–H stretching peak at 3313 cm^–1^ in the neat PVA hydrogel shifts to 3288 cm^–1^ in spectrum (ii), which can be attributed to the overlapping contribution of N–H stretching vibrations from chitosan. With increasing chitosan content, the corresponding band in spectrum (iii) shifts slightly to 3296 cm^–1^, indicating enhanced interactions between PVA and chitosan chains. The progressive broadening of this band with increasing chitosan ratio suggests intensified intermolecular interactions, mainly hydrogen bonding between the two polymers.^32^ The peaks observed at 2952 and 2953 cm^–1^ in spectra (ii) and (iii) are assigned to CH_2_ stretching vibrations of the methyl groups. The absorption bands at 1653 and 1658 cm^–1^ are characteristic of N–H bending vibrations associated with amide groups in the chitosan structure. Due to the presence of aliphatic amino groups in chitosan, the sharp C–O–C stretching peak of PVA at 1094 cm^–1^ shifts to 1088 and 1079 cm^–1^ in spectra (ii) and (iii), respectively. Similarly, the CH_2_ bending peak at 1420 cm^–1^ in spectrum (i) shifts to approximately 1410 cm^–1^ in the PVA/CS hydrogels, reflecting the influence of chitosan incorporation.^33^ The overlapping and shifting of characteristic peaks indicate the formation of a well-integrated composite structure with strong molecular-level interactions. Overall, the FT-IR results confirm that PVA and chitosan are successfully combined to form IPN-like PVA/CS hydrogels with effective interpolymer interactions.

**Figure 1:**
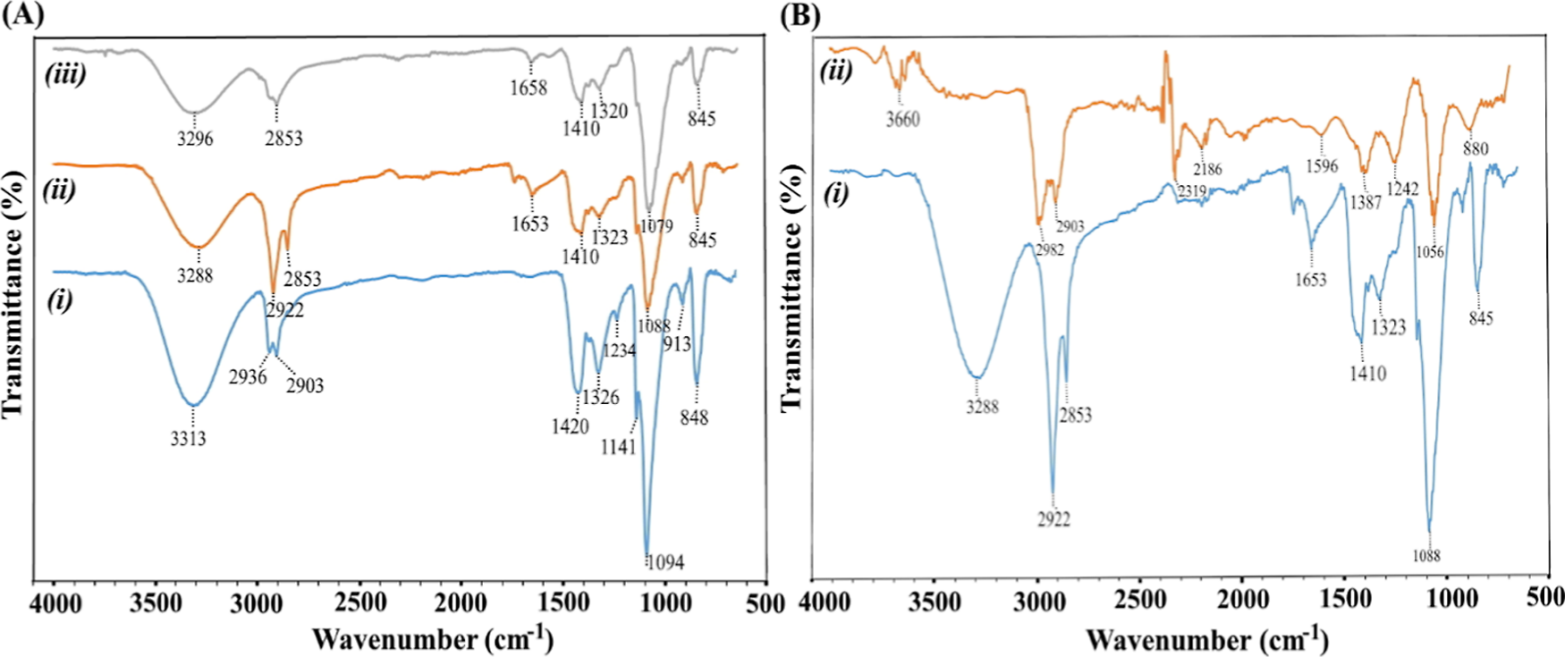
FT-IR spectra of PVA/CS hydrogels. (A) Hydrogels containing different ratios of CS in their structure: (i) C_1_P_0_, (ii) C1P30, and (iii) C_1_P_50_. (B) Before and after degradation at pH_2_: (i) C_1_P_30_ and (ii) C_1_P_30_pH_2_.

Figure 1B shows the FT-IR spectra of the C_1_P_30_ hydrogel (i) and the same hydrogel after immersion in a pH 2 solution for 21 days (C1P30pH2), (ii). A comparison of these spectra reveals pronounced changes in the chemical structure of the hydrogel after exposure to an acidic environment. It is well documented that degradation of PVA/CS hydrogels in acidic media can induce specific alterations in FT-IR spectra due to changes in functional groups and bond structures.^34,35^ In the present case, spectrum (i) exhibits relatively regular and well-defined absorption bands, whereas spectrum (ii) shows reduced peak intensities, peak shifts, the emergence of new bands, and overall spectral irregularities. Notably, the band at 3288 cm^–1^ in spectrum (i), associated with O–H stretching and PVA–CS interactions, shifts to 3660 cm^–1^ in spectrum (ii). This shift is indicative of hydroxyl group dissociation, hydrolysis processes, and the weakening of intermolecular interactions between PVA and chitosan chains. Furthermore, the aliphatic C–H stretching peaks at 2922 and 2853 cm^–1^ observed in spectrum (i) show attenuation and slight shifts in spectrum (ii), suggesting polymer chain scission during degradation. In acidic conditions, protonation of chitosan amino groups leads to the formation of –NH_3_^+^ species, which is reflected by the shift of the N–H bending vibration peak from 1653 cm^–1^ to 1596 cm^–1^.^12,36^ Taken together, these spectral changes provide clear evidence that the PVA/chitosan hydrogel undergoes structural degradation in acidic environments, consistent with its pH-sensitive behavior.

Figure 2 presents SEM images of PVA/CS hydrogel samples before and after degradation under different pH conditions, illustrating the combined effects of pH, chitosan content, and the freeze–thaw (F–T) cycle number on surface morphology and structural stability. As observed in the figure, the C_1_P_0_ hydrogel, which does not contain chitosan and was synthesized by using a single F–T cycle, exhibits a relatively homogeneous structure with large and open pores. In contrast, the C_4_P_0_ hydrogel with the same composition but subjected to four F–T cycles shows a much denser morphology characterized by smaller and more compact pores. This change can be attributed to the increased number of physical cross-linking points formed between PVA chains as the number of F–T cycles increases, leading to a tighter polymer network. With increasing cycle numbers, the initially large pore structures progressively shrink, resulting in a more compact morphology after multiple cycles. A similar trend is observed in hydrogels containing a 50% chitosan solution. The C_1_P_50_ hydrogel synthesized with a single F–T cycle displays a highly porous and heterogeneous structure with relatively large pores. However, the C_2_P_50_ and C_3_P_50_ hydrogels, prepared with two and three F/T cycles, respectively, exhibit reduced pore sizes and a more homogeneous surface morphology. The C_4_P_50_ hydrogel, subjected to four F–T cycles, shows the densest structure among the chitosan-containing samples, which can be explained by the formation of a higher number of physical interactions between PVA segments and chitosan chains. The incorporation of chitosan into the polymer network introduces additional interaction sites, resulting in a more complex network architecture compared to that of pure PVA hydrogels. For instance, when comparing the C_1_P_0_ and C_1_P_50_ samples synthesized with the same number of F–T cycles, the presence of chitosan in the C_1_P_50_ hydrogel leads to increased porosity, larger pore sizes due to structural heterogeneity, and a more irregular porous surface.^37^ Degradation studies conducted in PBS solutions with different pH values further reveal the influence of the medium on the hydrogel structural stability. The degradation of the pure PVA hydrogel C_1_P_0_ in a pH 2 environment (C_1_P_0_pH_2_) was calculated to be approximately 13%. SEM observations indicate that despite minor changes in surface roughness and average pore diameter, no significant morphological alterations occurred. This behavior can be attributed to the protonation of hydrophilic hydroxyl groups on PVA segments under acidic conditions, which reduces swelling and consequently enhances resistance to degradation. In contrast, the C_1_P_50_ hydrogel, which contains 50% chitosan and was synthesized with the same number of F–T cycles, exhibits pronounced morphological changes after degradation in a pH 2 environment (C_1_P_50_pH_2_). The initially large-pore structure transforms into a smaller-pore and denser morphology. Degradation tests indicate a degradation rate of approximately 38% for this sample, suggesting that the observed morphological changes are strongly influenced by the presence of chitosan. The partial dissolution of chitosan under acidic conditions and its diffusion from the pore structure into the surrounding medium likely contribute to pore filling and structural collapse. In addition, the reduction in swelling behavior under acidic conditions further promotes the formation of a denser network.

**Figure 2:**
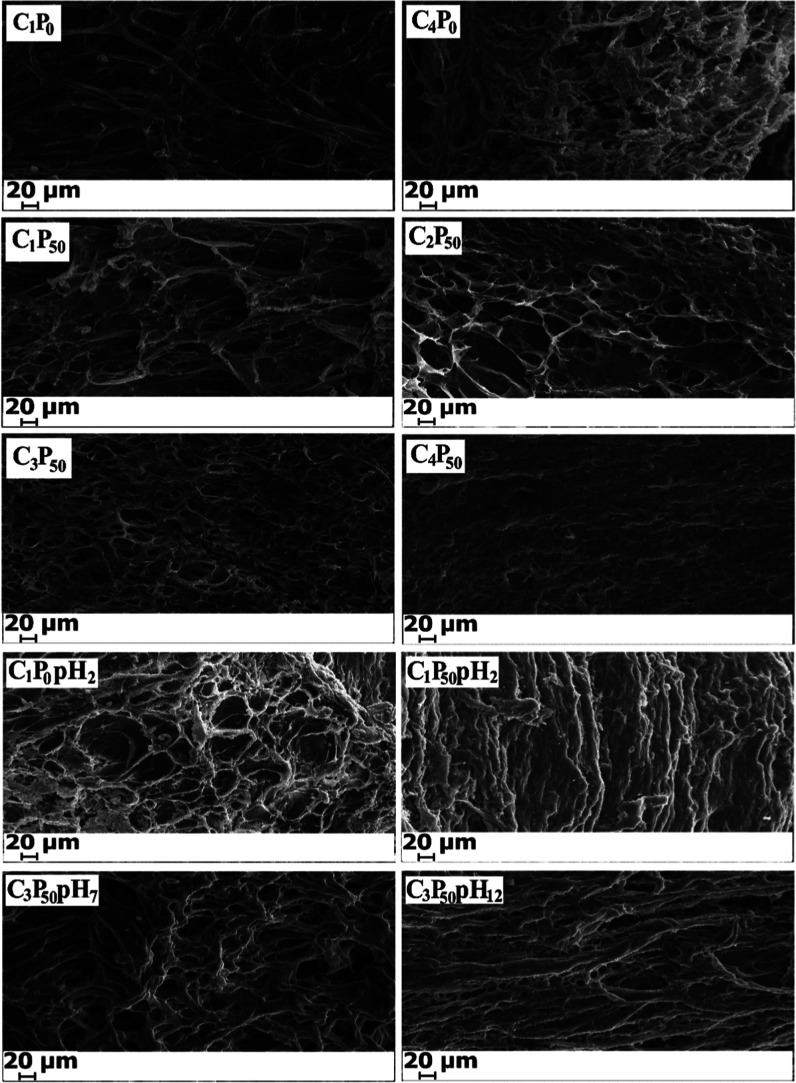
SEM images of PVA/CS hydrogels before and after degradation at different pH conditions.

In neutral environments, degradation effects on the hydrogel morphology are relatively limited. For example, the C_3_P_50_ hydrogel exhibits a degradation rate of approximately 12%, with SEM images showing partial embedding of pores rather than severe structural disruption. This effect becomes slightly more noticeable with increasing F–T cycle numbers but does not lead to major morphological changes. Similarly, examination of the C_3_P_50_ hydrogel degraded at pH 12 (C_3_P_50_pH_12_) revealed that the porous structure largely maintains its integrity under these conditions. Although the overall degradation rates of PVA/CS hydrogels remain low in basic environments, noticeable changes in surface morphology are still observed. For instance, the degradation rate of the C_3_P_50_ hydrogel at pH 12 is approximately 5%, yet SEM images show a transition from a homogeneous pore distribution to a rougher and denser surface. This behavior can be explained by the reduction in swelling values under basic conditions, which induces shrinkage of the hydrogel matrix and results in a denser and more rigid structure. Overall, degradation is most pronounced under acidic conditions, where pore structures become increasingly irregular and brittle, particularly in hydrogels with a higher chitosan content. In contrast, structural degradation is less significant at neutral pH, while in basic environments, the pore structure remains comparatively more regular and stable.

Taken together, the SEM observations demonstrate that pH, chitosan concentration, and freeze–thaw cycle number collectively govern the morphology, structural stability, and controlled degradation behavior of PVA/CS hydrogels, highlighting the importance of multiparameter design in tailoring these materials for biomedical applications.

### Swelling Studies

Swelling experiments of the PVA/CS hydrogel composites were carried out in phosphate-buffered saline (PBS) solutions with different pH values at 37 °C in order to investigate the effects of chitosan concentration and the F–T cycle number on swelling behavior. Based on these parameters, representative swelling kinetic curves of selected hydrogels are presented in Figure 3, while the swelling profiles of all hydrogel composites are provided in Figures S1–S4. The swelling experiments were performed in triplicate for each sample to ensure reproducibility. Data points in Figure 3 represent the mean values of these three independent measurements, with error bars indicating the standard deviation (SD). The narrow distribution of the error bars confirms the consistency of the swelling behavior across the tested PVA/CS hydrogel formulations. The obtained plots clearly illustrate the time-dependent swelling behavior of the PVA/CS hydrogels. In general, most hydrogels reached values close to their equilibrium swelling within the first 3 days of incubation. It can be stated that parameters such as cross-linking density, type and concentration of functional groups, network structure, and environmental pH play key roles in determining the swelling characteristics of the hydrogel systems.

**Figure 3:**
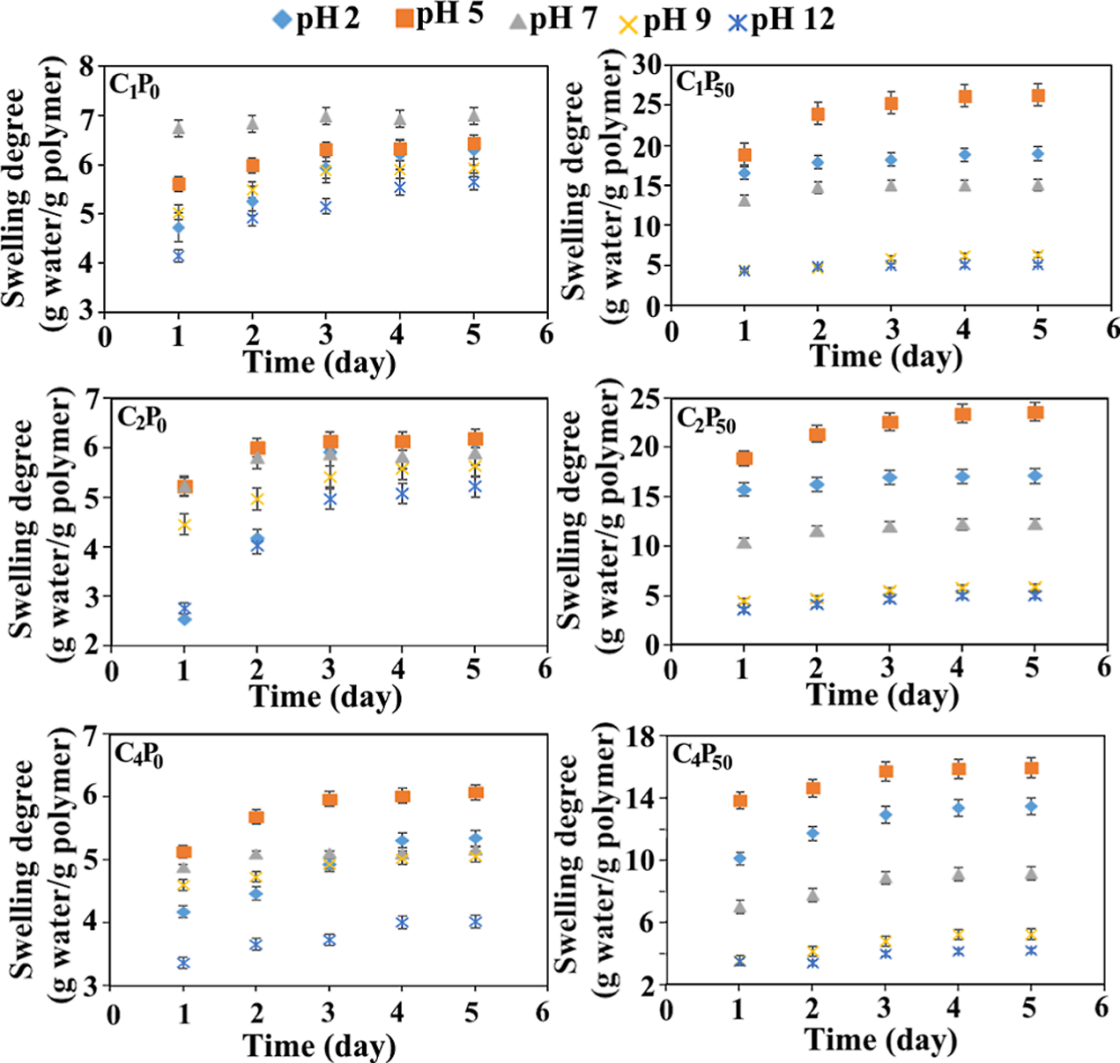
Swelling kinetic curves of PVA and PVA/CS hydrogels synthesized using single, two, and four freeze–thaw cycles.

Analysis of the swelling kinetics curves indicates that pure PVA hydrogels without chitosan exhibit relatively low swelling capacities at all investigated pH values, ranging approximately between 4 and 7 g of water/g of polymer. These swelling behaviors are influenced by both pH and, to some extent, the number of F–T cycles applied during synthesis. For pure PVA hydrogels prepared with a single F-to-T cycle, the highest swelling ratio was observed under neutral conditions (pH 7) compared to other pH levels. This behavior can be attributed to the hydrophilic nature of PVA and the presence of abundant hydroxyl (–OH) groups in its structure, which are able to form strong hydrogen bonds with water molecules under neutral pH conditions.^37^ In the absence of significant protonation or deprotonation effects at neutral pH, these hydroxyl groups remain highly effective in attracting water molecules, thereby enhancing the swelling capacity of the hydrogel.^38,39^

In contrast, pure PVA hydrogels synthesized with two or more F–T cycles exhibit their maximum swelling values at pH 5 rather than at pH 7. In hydrogels with higher freeze–thaw cycle numbers, swelling behavior is mainly governed by hydrogen bonding interactions and the balance between crystalline and amorphous regions, rather than by ionic effects. Under mildly acidic conditions, partial weakening of interchain hydrogen bonds increases polymer chain mobility and facilitates water diffusion, while the crystalline junction zones formed during freeze–thaw processing remain largely preserved. This combined effect leads to enhanced swelling at pH 5.^40,41^

At strongly acidic conditions such as pH 2, the swelling ratios of pure PVA hydrogels remain lower compared to those observed at neutral pH. This behavior can be explained by protonation of hydroxyl (–OH) groups, which limits their ability to form hydrogen bonds with water molecules and consequently reduces water uptake. Similarly, in basic pH environments, hydroxyl groups tend to carry a negative character, and the osmotic pressure difference between the polymer network and the surrounding medium becomes minimal, resulting in reduced water absorption. As a result, the swelling capacity of PVA hydrogels decreases at pH values away from neutrality.^41^

Evaluation of the swelling kinetic curves reveals that increasing the chitosan content in the hydrogel structure leads to a pronounced increase in the equilibrium swelling values of the PVA/CS hydrogels in acidic media. For instance, the equilibrium swelling values at pH 5 for the C_1_P_0_, C_1_P_30_, and C_1_P_50_ hydrogels synthesized with a single freeze–thaw (F–T) cycle were determined to be 6.44, 14.6, and 26.27 g water/g polymer, respectively. This behavior can be explained by the protonation of amino (–NH_2_) groups in the chitosan structure under low pH conditions, resulting in positively charged –NH_3_^+^ groups. The electrostatic repulsion between these charged groups increases the free volume between polymer chains and promotes network expansion, allowing water molecules to penetrate the hydrogel matrix more easily. As a result, both the swelling rate and the equilibrium swelling capacity increase. In addition, protonated amino groups interact more strongly with water molecules due to enhanced electrostatic attractions, further contributing to the increased swelling behavior.^42^

In general, PVA/CS hydrogels exhibit higher swelling values at pH 5 compared with those observed at pH 2. For example, the swelling values of the C_2_P_50_ hydrogel at pH 2 and pH 5 were measured as 17.12 and 23.65 g water/g polymer, respectively. This difference can be attributed to the reduced osmotic pressure gradient between the polymer network, containing positively charged –NH_3_^+^ groups, and the surrounding medium with a high concentration of H^+^ ions under strongly acidic conditions. In neutral and basic environments, the swelling values of all PVA/CS hydrogels remain relatively low. This behavior arises from the deprotonation of amino groups (–NH_2_) in the chitosan segments, leading to the loss of positive charges and weakening of electrostatic interactions. Consequently, interactions between the hydrogel network and water molecules decrease, resulting in a lower swelling capacity.^41,43^ Across all hydrogel series, an increase in the number of F–T cycles leads to a systematic decrease in equilibrium swelling values. For example, the equilibrium swelling values at pH 5 for C_1_P_50_, C_2_P_50_, C_3_P_50_, and C_4_P_50_ hydrogels synthesized with one, two, three, and four F–T cycles were found to be 26.27, 23.01, 18.09, and 15.90 g of water/g of polymer, respectively. According to the literature, increasing the number of F–T cycles enhances the cross-linking density within the hydrogel network. A higher cross-linking density results in a more compact polymer structure, which restricts the diffusion of water molecules into the hydrogel matrix and thereby reduces the overall swelling capacity.^43^

### Degradation Studies

Degradation of hydrogels in aqueous media initiates with penetration of the solution into the polymer network. Water molecules diffuse into the internal structure of the hydrogel and interact with the bonds between polymer chains. In general, degradation in aqueous environments may occur through three main mechanisms, namely, hydrolysis, swelling-induced degradation, and pH-related effects. In hydrolytic degradation, water molecules react with relatively weak bonds within the polymer structure. Certain chemical bonds present in hydrogels, particularly hydrolysis-sensitive bonds, such as ester and amide linkages, gradually cleave upon interaction with water. This process leads to fragmentation of polymer chains into smaller segments and eventually contributes to the dissolution of the hydrogel over time. In degradation governed by swelling, the diffusion of water into the hydrogel matrix causes volumetric expansion of the network. Swelling increases the distance between polymer chains, resulting in a loosening of the structure. As a consequence, the mechanical integrity of the hydrogel decreases, making the material more susceptible to fragmentation. Hydrogels with relatively loose network structures or low cross-linking densities are therefore more prone to rapid degradation through this mechanism. In pH-dependent degradation, acidic or basic environments can alter the bonding characteristics of the polymer network, leading to accelerated breakdown. The rate and extent of degradation are closely related to the accessibility of water molecules to the polymer matrix.^44,45^ The percentage degradation values of the PVA/chitosan hydrogel series synthesized with different freeze–thaw cycle numbers and exposed to PBS solutions at various pH levels are presented in Figure 4.

**Figure 4:**
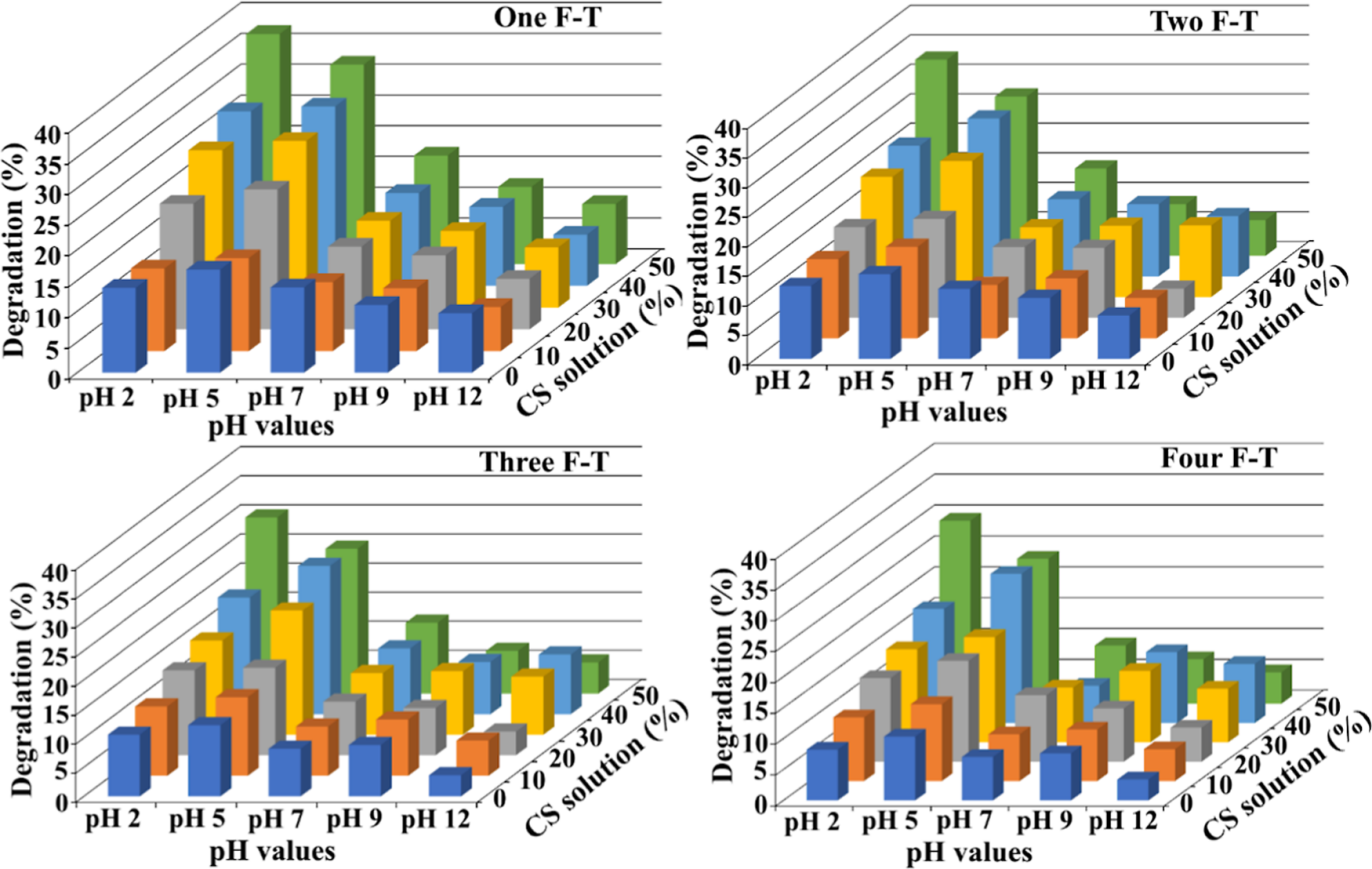
Degradation values of PVA/CS hydrogels in PBS at 37 °C.

Degradation tests were similarly conducted in triplicate, and the results are presented as mean values in Figure 4. It is noteworthy that the standard deviations for all degradation data points were found to be less than 3%, indicating a high experimental precision. For the sake of visual clarity in the bar charts, these minimal error bars were omitted; however, the low variability underscores the stability of the degradation process under the specified conditions. Examination of these 3D plots reveals a general correlation between the degradation behavior and the swelling capacities of the hydrogels. It is evident that hydrogel degradation, similar to swelling behavior, is more pronounced in acidic environments compared to neutral and basic conditions. For instance, the C_1_P_30_ hydrogel exhibits degradation values of 25.52%, 23.05%, 14.09%, 12.43%, and 7.79% at pH 2, 5, 7, 9, and 12, respectively. This trend can be explained by the tendency of chitosan-based hydrogels to undergo dissolution under acidic pH conditions. As chitosan is a weak base, it becomes protonated and more soluble in aqueous media at pH values below approximately 6.

In acidic environments, the protonation of amino groups in the chitosan segments generates positive charges within the polymer structure. This increases electrostatic repulsion forces, leading to the enlargement of the interchain spacing and expansion of the polymer network. As a consequence, the swelling behavior of the hydrogel is enhanced, allowing water molecules to penetrate the structure more effectively.^46,47^ The resulting network expansion weakens the physical interactions within the polymer matrix, reducing interchain cohesion and ultimately decreasing the mechanical stability and structural integrity. Under neutral conditions, the amino groups of chitosan remain largely unprotonated, resulting in weaker electrostatic interactions. This leads to lower swelling and a reduced degradation rate compared with acidic environments. The hydrogel structure therefore exhibits a greater resistance to water penetration and degradation under neutral pH conditions. Furthermore, the degradation plots indicate that degradation decreases progressively with increasing alkalinity of the medium. In basic environments, deprotonation of amino groups induces negative charges along the polymer chains, which promote electrostatic interactions that hinder diffusion of water into the polymer network. Consequently, the swelling ratio decreases, which helps to preserve the structural integrity of the hydrogel. As the pH of the medium increases further, the osmotic pressure difference across the hydrogel network diminishes, resulting in reduced swelling and, in turn, lower degradation values.^48^ Overall, the observed differences in degradation behavior under acidic, neutral, and basic conditions arise from variations in water accessibility to the polymer network and the pH-responsive nature of the hydrogel system.

Analysis of the 3D plots indicates a direct relationship between the chitosan content and degradation rate. For example, the degradation values of the chitosan-free C_1_P_0_ hydrogel at pH 2, 7, and 12 were determined to be 13.80, 13.87, and 5.68%, respectively, whereas the corresponding degradation values of the C_1_P_50_ hydrogel at the same pH levels were 37.36, 17.61, and 9.74%. As can be seen, the difference in degradation due to chitosan incorporation is more pronounced under acidic conditions, while it becomes relatively lower in neutral and basic environments. With increasing chitosan content, the swelling capacity of the hydrogel increases as a result of higher osmotic pressure and stronger electrostatic repulsion among protonated –NH_3_^+^ groups, leading to a looser network structure and, consequently, higher degradation rates. This behavior can be associated with the structural characteristics of the polymer network as well as the strong hydrogen bonding interactions involving –NH_2_ and –OH groups present in the chitosan structure.^49^ Literature reports indicate that hydrogels composed solely of PVA tend to form more stable crystalline regions between polymer chains. These crystalline domains arise from the regular folding and alignment of PVA chains and contribute to the enhanced mechanical strength and rigidity of the material. Consistent with these findings, the degradation plots show that hydrogels coded as P0, which do not contain chitosan, exhibit lower degradation levels. The absence of an ionically charged copolymer such as chitosan results in a tighter polymer network, making pure PVA hydrogels more resistant to structural breakdown and degradation.^50^

Optimization of the number of F–T cycles plays a critical role in controlling both the swelling kinetics and degradation rate of hydrogel systems. As observed in Figure 5, an increase in the number of F–T cycles results in a gradual decrease in degradation rates. This behavior can be explained by the formation and interconnection of crystalline regions generated during each successive cycle. With increasing F–T cycle numbers, the number of crystalline domains formed within the polymer network increases, and these domains act as physical cross-linking points, leading to a stiffer and more rigid hydrogel structure. As a result, the swelling capacity of the hydrogel decreases, which in turn contributes to a reduction in the degradation rate. For example, in the C_2_ hydrogel series, the application of one additional F–T cycle compared to the C_1_ series leads to the formation of a denser hydrogel network. Consequently, the degradation rates of the C_2_ hydrogels were found to be relatively lower than those of the C_1_ series. While the effect of the F–T cycle number on degradation is pronounced in hydrogels with low chitosan content, this effect becomes less significant as the chitosan concentration in the structure increases. For instance, the degradation rate of the chitosan-free P_0_C_1_ hydrogel synthesized with a single F–T cycle was measured as 16.76% at pH 5, whereas the degradation rate of the P_0_C_4_ hydrogel synthesized with four F–T cycles under the same conditions decreased to 10.31%. This corresponds to an approximate reduction of 38.5% in degradation after the fourth cycle. In contrast, for hydrogels containing 50% chitosan, the degradation rate of the P50C1 hydrogel at pH 5 was 27.37% while that of the P50C4 hydrogel decreased to 23.50%, corresponding to a reduction of approximately 14.14% after four F–T cycles. These results indicate that as the chitosan content in the hydrogel increases, the influence of the F–T cycle number on degradation behavior diminishes. This phenomenon can be attributed to the suppression of crystalline region formation per unit volume during the F–T process due to the incorporation of chitosan into the polymer network. Since crystalline domains formed during F–T cycling predominantly originate from PVA segments, the presence of chitosan interferes with PVA crystallization, reduces interactions between crystalline regions, and ultimately lowers the effective cross-linking density of the hydrogel. As a consequence of reduced cross-linking density, the structural stability of the hydrogel decreases, leading to increased swelling and, accordingly, higher degradation rates.

**Figure 5:**
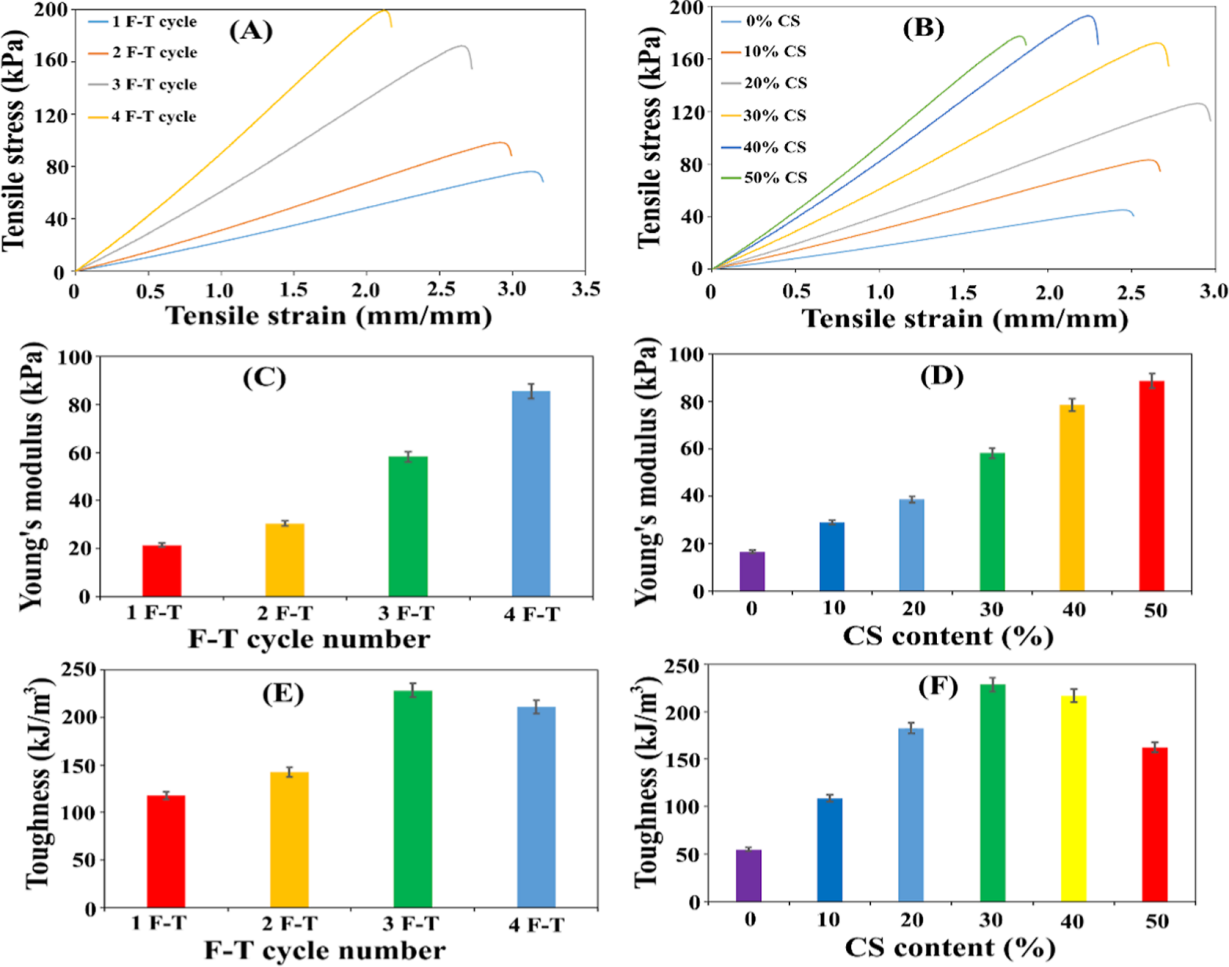
Mechanical characterization of PVA/CS hydrogels: (A) stress–strain curves of hydrogels containing a 30% CS solution as a function of the F–T cycle number; (B) stress–strain curves of hydrogels synthesized with 3 F–T cycles as a function of CS concentration; (C) Young’s modulus and (E) toughness values for hydrogels with varying F–T cycles (fixed 30% CS); (D) Young’s modulus and (F) toughness values for hydrogels with varying CS content (fixed 3 F–T cycles).

### Mechanical Studies

The mechanical tensile profiles, Young’s modulus values, and toughness properties of hydrogels synthesized with a fixed 30% chitosan (CS) solution across varying freeze–thaw (F–T) cycles are presented in Figures 5A,C,E, respectively. Furthermore, the corresponding mechanical parameters for hydrogels containing diverse CS concentrations subjected to three fixed F–T cycles are illustrated in Figures 5B,D,F.

As depicted in Figure 5A, the number of F–T cycles exerts a profound influence on the elongation and tensile strength of the hydrogels. The C_1_P_30_ hydrogel (1 F–T cycle) exhibited a maximum elongation of 3.12 mm/mm and a tensile strength of 76.38 kPa. In contrast, the C_4_P_30_ hydrogel (4 F–T cycles) demonstrated a reduced maximum elongation of 2.11 mm/mm and a significantly enhanced tensile strength of 199.58 kPa. The observed decrease in extensibility with increasing number of F–T cycles is attributed to the heightened cross-linking density. The densification of the network architecture restricts the sliding motion of polymer chains, thereby reducing the material’s flexibility. Conversely, the increase in mechanical strength is explained by the progressive integration of polymer chains into the crystalline lattice with each subsequent cycle, which enhances the rigidity of the gel. This increased crystallinity yields a more robust network that improves resistance to deformation.^51^ Corroborating these findings, Figure 5C reveals that the Young modulus values—ranging from 21.44 to 85.60 kPa—exhibited a positive correlation with the number of F–T cycles, further indicating the formation of a more compact network structure. Notably, the toughness values (Figure 5E) increased up to three F–T cycles (from 117.8 to 228.52 kJ/m^3^) before declining to 211.13 kJ/m^3^ in the fourth cycle. This peak at the third cycle (228.52 kJ/m^3^) followed by a decline is a critical observation, suggesting that the energy dissipation capacity diminishes beyond a certain threshold due to excessive brittleness. For PVA/CS hydrogels containing 30% CS, the third F–T cycle represents the optimum synergy, providing an ideal balance between ductility and strength.

Figure 5B demonstrates that the chitosan content significantly modulates the mechanical properties of the PVA/CS hydrogels. While the CS-free hydrogel (C_3_P_0_) showed a maximum elongation of 2.45 mm/mm and a strength of 45.07 kPa, the C_3_P_30_ hydrogel (30% CS) exhibited superior values of 2.66 mm/mm and 172.48 kPa, respectively. Elongation was found to increase with CS content up to 20% (reaching 2.94 mm/mm) but subsequently declined to 1.85 mm/mm. The initial increase suggests that at lower concentrations, CS may act as a “plasticizer” within the PVA matrix, facilitating chain mobility. However, the sharp decline beyond 20% CS is attributed to the dominance of rigid CS chains, which leads to comprehensive matrix stiffening. The tensile strength increased progressively with CS content up to 40% (from 45.07 to 193.17 kPa) before a slight reduction at 50% CS (177.28 kPa). According to Figure 5D, Young’s modulus values (16.53–88.70 kPa) followed a similar upward trend. This continuous enhancement in strength and modulus up to 40% of CS is governed by the dense hydrogen bonding networks formed between the amino (–NH_2_) groups of CS and the hydroxyl (–OH) groups of PVA. In this configuration, CS acts as a rigid backbone within the matrix, facilitating efficient stress transfer. The slight decrease observed at 50% CS may be linked to the onset of CS molecule aggregation and the disruption of PVA crystalline domains (phase separation).^52^ As shown in Figure 5F, toughness reached a maximum at 30% CS (228.52 kJ/m^3^). This peak signifies the composition where interpolymer interactions are most efficient, allowing the material to absorb maximum energy before fracture.

Although the present study focuses on the content optimization of PVA/CS hydrogels, their biological safety is a critical factor for clinical applicability. The freeze–thaw (F–T) method employed in this synthesis is a physical cross-linking technique that circumvents the use of potentially toxic chemical cross-linkers or organic solvents, which are often associated with cellular toxicity. Extensive literature on F–T-processed PVA/CS systems has consistently demonstrated their noncytotoxic nature, excellent cell viability, and hemocompatibility, confirming their safety for contact with biological tissues.^14,52–55^ The established mechanical properties indicate that these synthesized PVA/CS hydrogels possess substantial potential for biomedical applications. Specifically, the high toughness and tunable Young’s modulus suggest that these hydrogels can fulfill the specific mechanical requirements of various internal tissues, making them excellent candidates for tissue engineering scaffolds,^53^ wound dressings,^54^ and controlled drug delivery systems.^55^

### Modeling of the Swelling and Degradation Behaviors of PVA/CS Hydrogels

Conventional kinetic models, such as zero-order, first-order, pseudo-second-order, and the Korsmeyer–Peppas equations (Table S1), are fundamentally designed to delineate the influence of a restricted set of variables through linear or simplified exponential functions.^56,57^ Due to inherent limitations—namely, the omission of synergistic effects, linearization biases, mechanical constraints, and the inability to account for dynamic environments—these models often fail to capture the complexity of real-world data, providing only a unidirectional mechanical interpretation.^17,58^ The swelling and degradation dynamics of PVA/CS hydrogel systems are governed by the simultaneous and nonlinear interplay of multiple parameters, including pH, polymer composition, chitosan content, F–T cycle frequency, and temporal factors. Modeling such multifaceted systems solely through traditional frameworks presents significant hurdles regarding time efficiency, cost-effectiveness, and overall model fidelity. Unlike classical approaches, artificial neural networks (ANN) bypass the requirement for predefined physical laws regarding system functionality; instead, they discern latent patterns within the data sets. Consequently, this study utilizes an advanced data-driven ANN methodology to model the swelling and degradation behaviors of synthesized PVA/CS hydrogels.

It is important to emphasize that the selected input parameter ranges (pH, chitosan content, and freeze–thaw cycle number) were deliberately chosen within the most frequently investigated and practically relevant intervals reported in the literature for PVA/chitosan hydrogel systems. In previous studies, PVA/CS hydrogels have typically been evaluated under acidic, neutral, and mildly basic environments, commonly within a pH range of approximately 2–12, to assess their pH-responsive swelling and degradation behavior relevant to biomedical applications.^20,22,31^ Similarly, freeze–thaw (F–T) cycle numbers are generally limited to 1–4 cycles, as several reports indicate that beyond four cycles, further increases in crystallinity and cross-linking density result in marginal changes in swelling behavior while increasing processing time.^59,60^ Moreover, in physically cross-linked PVA/CS systems prepared via F–T processing, chitosan content is commonly restricted to ≤50%, since higher CS ratios tend to disrupt PVA crystalline domains and compromise structural stability.^14,61^ Therefore, the parameter space adopted in this study not only encompasses a broad experimental range but also remains fully aligned with the practical and structurally stable design window established in the current PVA/CS hydrogel literature.

For swelling behavior modeling, the developed ANN architecture incorporated four main input parameters: time, pH, chitosan ratio, and number of F–T cycles. The selection of these parameters enabled a comprehensive representation of the physical and chemical mechanisms directly affecting the hydrogel network structure. The network consisted of a hidden layer with 20 logarithmic sigmoid (logsig) neurons, while a linear (purelin) activation function was applied at the output layer to allow a continuous and precise prediction of swelling values. The model was trained using a total of 720 data points, and its overall structure is schematically illustrated in Figure 6a, where the network architecture and activation function distribution are clearly presented.

**Figure 6:**
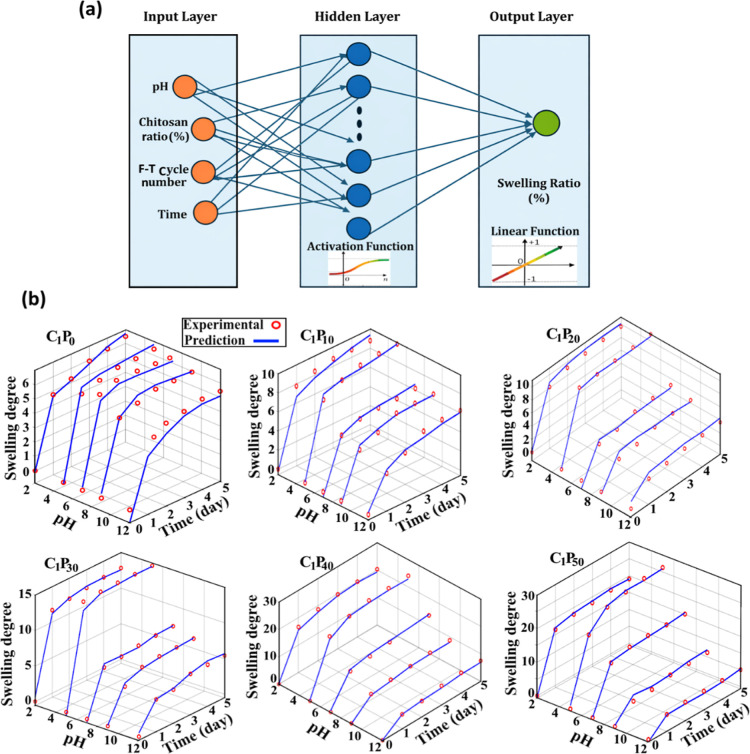
(a) Schematic representation of a generalized ANN structure created to predict the swelling value of hydrogels and (b) comparison of experimental and ANN model-predicted swelling values.

To quantitatively evaluate the model performance, several statistical indicators were employed, including the correlation coefficient (*R* ^2^), mean square error (MSE), root-mean-square error (RMSE), and mean absolute percentage error (MAPE). As summarized in Table 1, high correlation coefficients of *R* ^2^ = 0.9920 for the training data set and *R* = 0.9870 for the test data set were obtained. These values indicate a very strong agreement between the ANN predictions and experimental results, confirming the high generalization capability of the model. In addition, the low RMSE and MSE values observed for both training and test data sets demonstrate that the model not only fits the data well but also avoids overfitting. The MAPE values remaining within the range of approximately 3–4% further highlight that the swelling behavior can be predicted with high accuracy within an acceptable error margin for practical applications.

**Table 1:** Statistical Parameters of ANN Models Developed for Swelling and Degradation

	statistical parameters for swelling				statistical parameters for degradation			
training	*R* ^2^	RMSE	MSE	MAPE	*R* ^2^	RMSE	MSE	MAPE
	0.9920	0.7451	0.4432	3.2154	0.9870	0.8286	0.5139	4.1165
test	*R* ^2^	RMSE	MSE	MAPE	*R* ^2^	RMSE	MSE	MAPE
	0.9870	0.7741	0.4894	3.3756	0.9823	0.9074	0.5311	4.3326

The three-dimensional (3D) plots presented in Figure 6b provide a comparative visualization of the experimental and ANN-predicted swelling behavior of PVA/CS hydrogels synthesized with a single F–T cycle (C_1_ series) and containing different chitosan ratios. In these plots, the time–pH–swelling relationship is represented as a continuous surface, and it is clearly observed that the ANN-predicted surfaces closely follow the experimental data. In particular, the increasing trend in swelling values observed under acidic pH conditions and at higher chitosan contents is successfully captured by the ANN model. This agreement indicates that the model effectively learned the chitosan-driven pH-sensitive swelling mechanism and was able to represent the underlying nonlinear interactions within the hydrogel system.

Another important contribution of the 3D plots is that they clearly demonstrate that swelling behavior is not governed by a single parameter but rather by the combined interaction of multiple variables. The gradual approach of swelling values toward equilibrium over time, the variation in network expansion rates with changes in pH, and the amplifying effect of the chitosan content on these processes are all reflected as physically meaningful trends on the ANN-generated surfaces. In this context, Figure 6b serves not only as a validation of the modeling approach but also as a valuable visual output that can be used as a decision-support tool in hydrogel design.

In the modeling of the swelling behavior of C_2_P_20_ and C_3_P_40_ hydrogels in different pH environments, the correlation (*R* ^2^) values showing the agreement between the most frequently used models in the literature and the ANN model we developed are given in Table S2. When the prepared comparison table is examined, it is seen that the correlation coefficients (*R* ^2^) of the traditional models are significantly lower compared to those of the ANN model. While standard models used in swelling data can only accurately represent a certain stage of the system or a certain pH range, the ANN model provided high accuracy (*R* ^2^ ≈ 0.99) for the entire pH (2–12) and the entire composition range.

The ANN-based approach developed to model the degradation behavior of the synthesized PVA/CS hydrogels considers the key parameters directly influencing the hydrogel network structure within a unified framework. The use of three primary input variables—pH, chitosan ratio, and F-to-T cycle number—allows the degradation mechanism to be represented in terms of both chemical effects (pH and ionic interactions) and physical effects (cross-linking density and network compactness). As schematically illustrated in Figure 7a, the ANN architecture includes a hidden layer with 10 logarithmic sigmoid (logsig) neurons, which provides sufficient flexibility to learn the behavior of this multiparameter and nonlinear system. The use of a linear (purelin) activation function in the output layer enables an accurate and continuous prediction of degradation percentages as a quantitative response.

**Figure 7:**
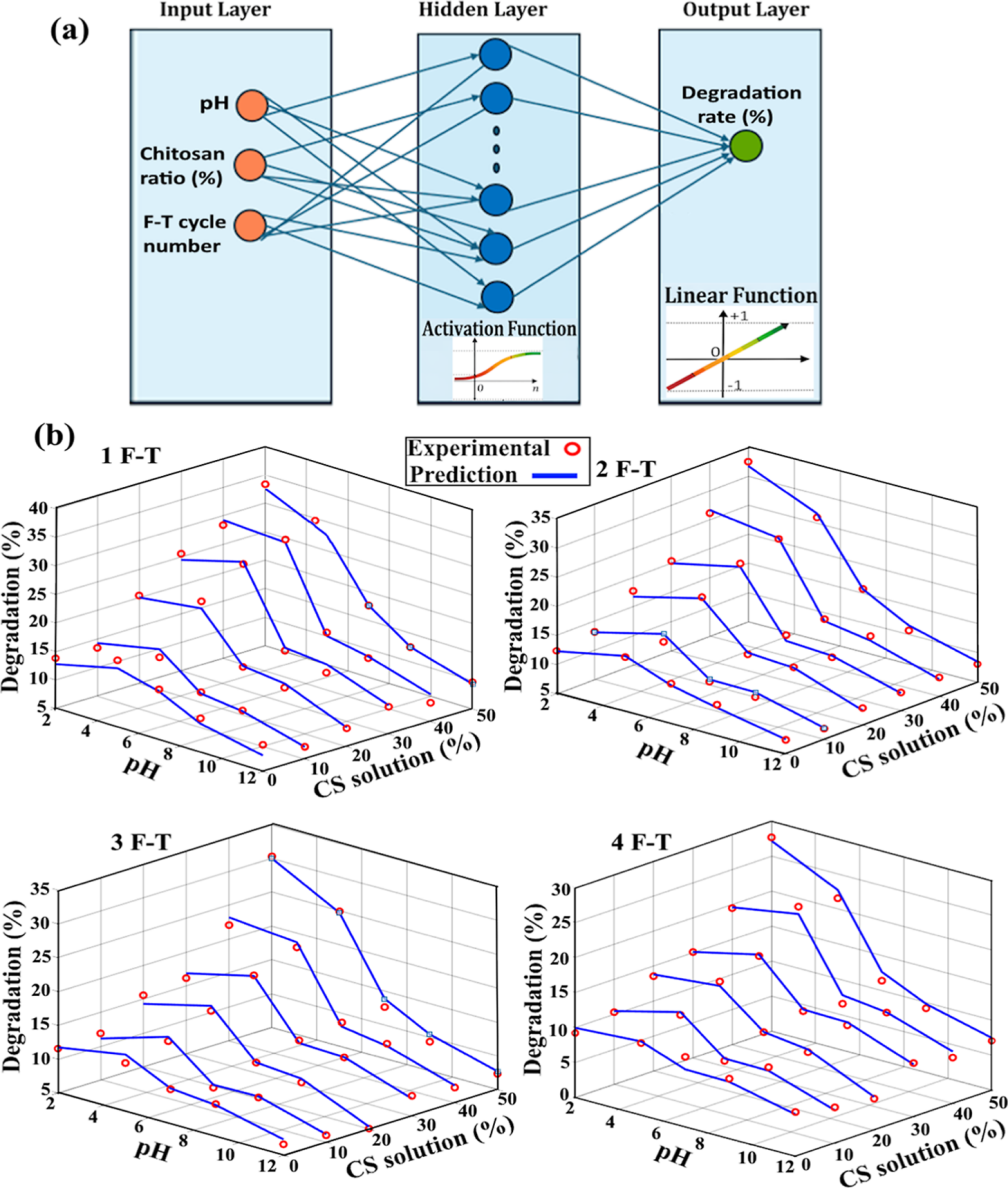
(a) Schematic representation of a generalized ANN structure created to predict the degradation value (%) of hydrogels and (b) comparison of experimental and ANN model-predicted degradation values.

The performance of the ANN model, evaluated using the statistical indicators summarized in Table 1, demonstrates highly satisfactory results. The correlation coefficients of *R* = 0.9864 for the training data set and *R* = 0.9823 for the test data set indicate a very strong agreement between the ANN predictions and the experimental degradation data. The close similarity between the training and test correlation values further confirms that the model exhibits a strong generalization capability rather than simply memorizing the training data. Moreover, the low RMSE and MSE values obtained for both data sets suggest that the model avoids overfitting and can predict degradation behavior in a stable and consistent manner. In particular, MAPE values within the range of approximately 4–5% highlight that the developed ANN model can estimate degradation rates with an acceptable level of error for practical applications, reinforcing its potential as a supportive decision-making tool in hydrogel design.

The 3D plots presented in Figure 7b visually illustrate the accuracy with which the ANN model represents the degradation behavior of the hydrogels. These plots show that the experimental and ANN-predicted degradation surfaces are distributed very closely across the parameter space defined by pH, chitosan content, and F–T cycle number. The pronounced increase in degradation values observed under acidic pH conditions and at higher chitosan ratios indicates that the ANN model successfully captured the chitosan-related pH-sensitive degradation mechanism. Under acidic conditions, protonation of amino groups in chitosan enhances electrostatic repulsion between polymer chains, leading to network loosening and facilitating water penetration into the hydrogel structure, which in turn accelerates degradation. The ability of the ANN-generated surfaces to accurately reflect this trend suggests that the model produces not only statistically reliable but also physically meaningful predictions.

Another key insight provided by the 3D degradation plots is that degradation behavior is governed by the interaction of multiple parameters rather than by a single variable. The overall decrease in degradation rates with increasing F–T cycle number can be attributed to the formation of additional crystalline regions and the resulting increase in physical cross-linking density within the PVA segments. In contrast, the partial suppression of this cycle-related effect at higher chitosan contents may be associated with the inhibition of PVA crystallization and the development of a more heterogeneous network structure. By visualizing these competing mechanisms on a single surface, the ANN-based 3D plots effectively clarify the complex nature of degradation behavior in PVA/CS hydrogels.

The utility of the developed ANN as a decision-support framework was rigorously verified through an unseen data paradigm. By isolating 15% of the total data set from the architectural optimization and weight-adjustment phases, this subset functioned as an autonomous blind-validation cohort. The high convergence between these strictly partitioned experimental observations and the network’s heuristic projections signifies a robust generalization capacity for interpolating hydrogel behavior under novel formulation-condition permutations. Within the computational modeling lexicon, such a methodology serves as a statistically sound surrogate for prospective validation, effectively mitigating the necessity for repetitive laboratory synthesis while reinforcing the model’s efficacy in preemptive material design.

It is imperative to underscore that the present study constitutes one of the most exhaustive implementations of ANNs specifically tailored for hydrogels synthesized via F–T processing to date. The swelling model—constructed by simultaneously integrating four critical variables (pH, copolymer composition, number of F–T cycles, and time)—alongside the degradation model—which incorporates three critical variables (pH, copolymer composition, and F–T cycles)—transcends the narrow parametric scope typically prevalent in the extant literature. The deliberate curation of a high-density data set not only justifies the robustness of architectural convergence but also enables the high-fidelity capture of complex, nonlinear synergistic effects governing hydrogel stability. This study establishes a robust benchmark for predictive material design in physically cross-linked systems, demonstrating how empirical data density can be strategically leveraged to achieve superior model generalizability.

Overall, the developed ANN model successfully predicted both the swelling and degradation behaviors of PVA/CS hydrogels with high accuracy, demonstrated strong agreement with experimental results, and effectively represented the relationships between pH sensitivity, composition, and physical cross-linking. This approach offers a powerful optimization and decision-support tool for reducing experimental workload, accelerating material design, and identifying hydrogel compositions suitable for targeted biomedical applications in multiparameter hydrogel systems.

## Conclusions

In this study, the structural characteristics, pH-responsive swelling behavior, and degradation performance of PVA/CS composite hydrogels synthesized via a freeze–thaw (F–T) method without chemical cross-linking were systematically investigated. The complex and nonlinear behaviors governing these properties were successfully modeled with high accuracy using artificial neural networks (ANNs), enabling a comprehensive evaluation of multiparameter hydrogel systems that are critical for biomedical applications. The experimental results demonstrated that chitosan content, environmental pH, and the number of F–T cycles play decisive roles in defining the hydrogel network architecture and, consequently, its swelling and degradation behavior. In acidic environments, the protonation of chitosan amino groups significantly enhanced electrostatic repulsion within the polymer network, leading to increased swelling and accelerated degradation. In contrast, increasing the number of F–T cycles promoted the formation of crystalline regions within the PVA segments, thereby increasing physical cross-linking density and resulting in a more compact and structurally stable hydrogel network. This structural reinforcement effectively reduced both swelling capacity and the degradation rate. The lower degradation observed in pure PVA hydrogels compared to chitosan-containing systems further highlights the dominant role of PVA crystalline domains in maintaining network stability. The ANN-based models exhibited excellent predictive performance, characterized by high correlation coefficients and low error values for both swelling and degradation behaviors. These results confirm that the ANN approach represents a robust and reliable modeling strategy for complex, multiparameter hydrogel systems. Notably, the three-dimensional ANN response surfaces provided physically meaningful representations of nonlinear parameter interactions, demonstrating the potential of this approach not only as a predictive tool but also as an effective decision-support framework for material design and optimization.

Overall, this study presents an integrated experimental and artificial-intelligence-based framework for the rational design of biocompatible, pH-sensitive, and tunable PVA/CS hydrogels. The findings offer valuable insights for the development of advanced hydrogel systems tailored for biomedical applications such as controlled drug delivery, wound dressing materials, and tissue engineering scaffolds. Future studies focusing on the integration of mechanical performance and biological responses into ANN models are expected to further advance the development of intelligent, application-oriented hydrogel design platforms.

## Supplementary Material



## Notes

Swelling kinetic curves of PVA and PVA/CS hydrogels synthesized with different freeze–thaw cycles (Figures S1–S4); kinetic model equations for drug release and swelling kinetics (Table S1); and correlation coefficient (*R* ^2^) values for swelling kinetics across different models (Table S2) (PDF)
