# Supplementary material for: Freeze–Thaw-Synthesized PVA/Chitosan Hydrogels: Structure–Property Relationships and ANN Modeling of Swelling and Degradation Behaviors
Source: ACS Omega. 2026 Apr 8;11(15):23385–400. doi: 10.1021/acsomega.6c00485 (PMC13103772; doi:10.1021/acsomega.6c00485)
Supplement: Supplementary file 1 [file ao6c00485_si_001.pdf]

Supporting Information

Freeze–Thaw Synthesized PVA/Chitosan Hydrogels: Structure–Property Relationships and ANN Modeling of Swelling and Degradation Behaviours

Elif İlayda Tarım[1], Cihangir Boztepe [1*], Mahmut Daşkın[2], Gozde Ozaydin İnce[3]

[1]*Department of Biomedical Engineering, Faculty of Engineering, Inonu University, 44280 Malatya, Türkiye*

[2]*Department of Mechanical Engineering, Faculty of Engineering, Inonu University, 44280 Malatya, Türkiye*

[3]*Department of Material Science and Nanoengineering, Faculty of Engineering and Natural Sciences, Sabanci University, Istanbul, 34956, Türkiye*

*Corresponding author: Cihangir Boztepe

E-mail address: cihangir.boztepe@inonu.edu.tr

Telephone : +90 422 377 4715

Fax : +90 422 377 4770

Contents

Figure S1. Swelling kinetic curves of PVA/CS hydrogels synthesized using a single freeze–thaw (F–T) cycle. S3

Figure S2. Swelling kinetic curves of PVA and PVA/CS hydrogels synthesized using two F–T cycles. S4

Figure S3. Swelling kinetic curves of PVA and PVA/CS hydrogels synthesized using three F–T cycles. S5

Figure S4. Swelling kinetic curves of PVA and PVA/CS hydrogels synthesized using four F–T cycles. S6

Table S1. Different kinetic model equations for drug release. S6

Table S2. Correlation coefficient (R^2) values of the swelling kinetics of hydrogels according to different models. S7

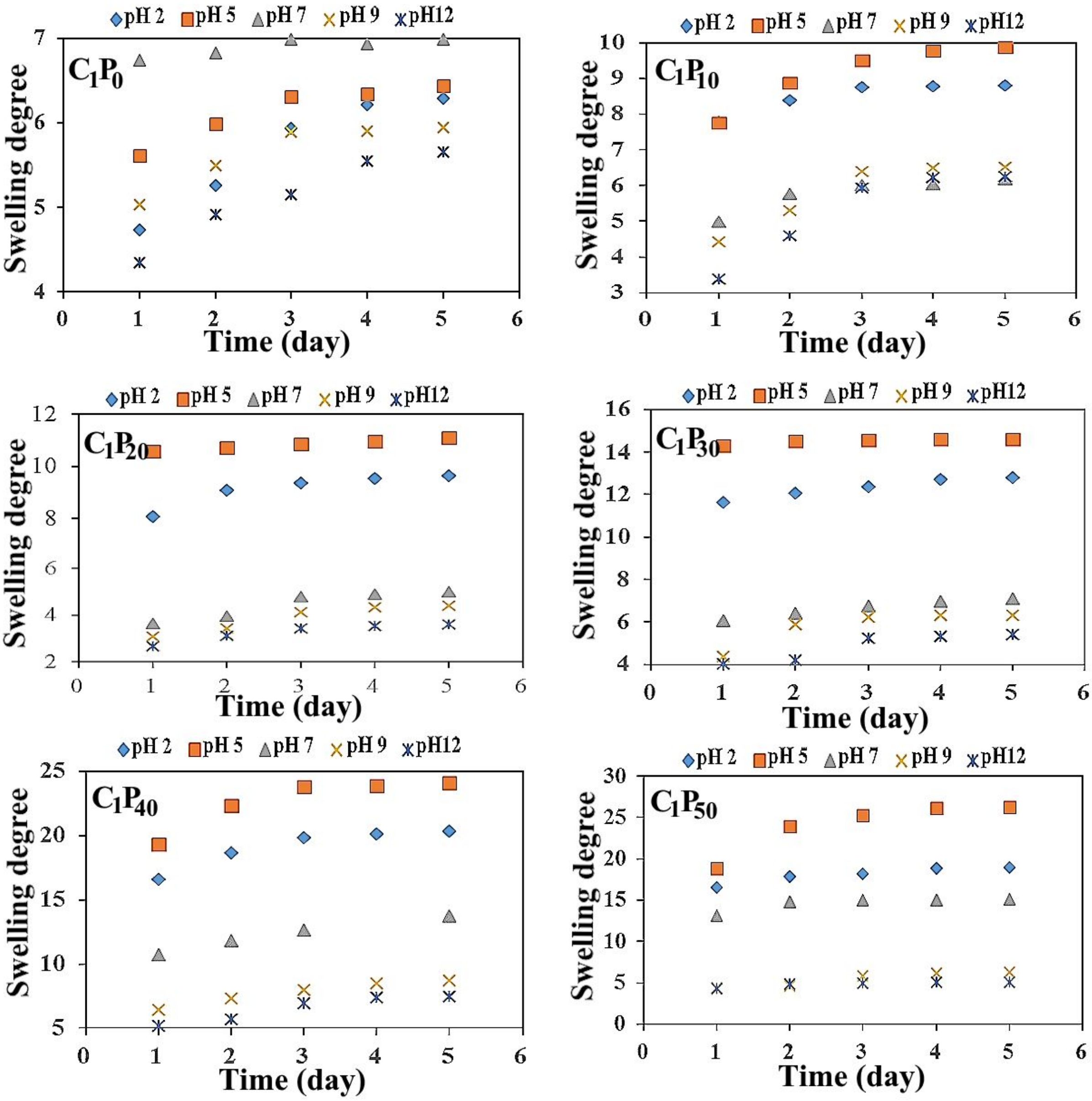

Figure S1. Swelling kinetic curves of PVA and PVA/CS hydrogels synthesized using a single freeze–thaw (F–T) cycle.

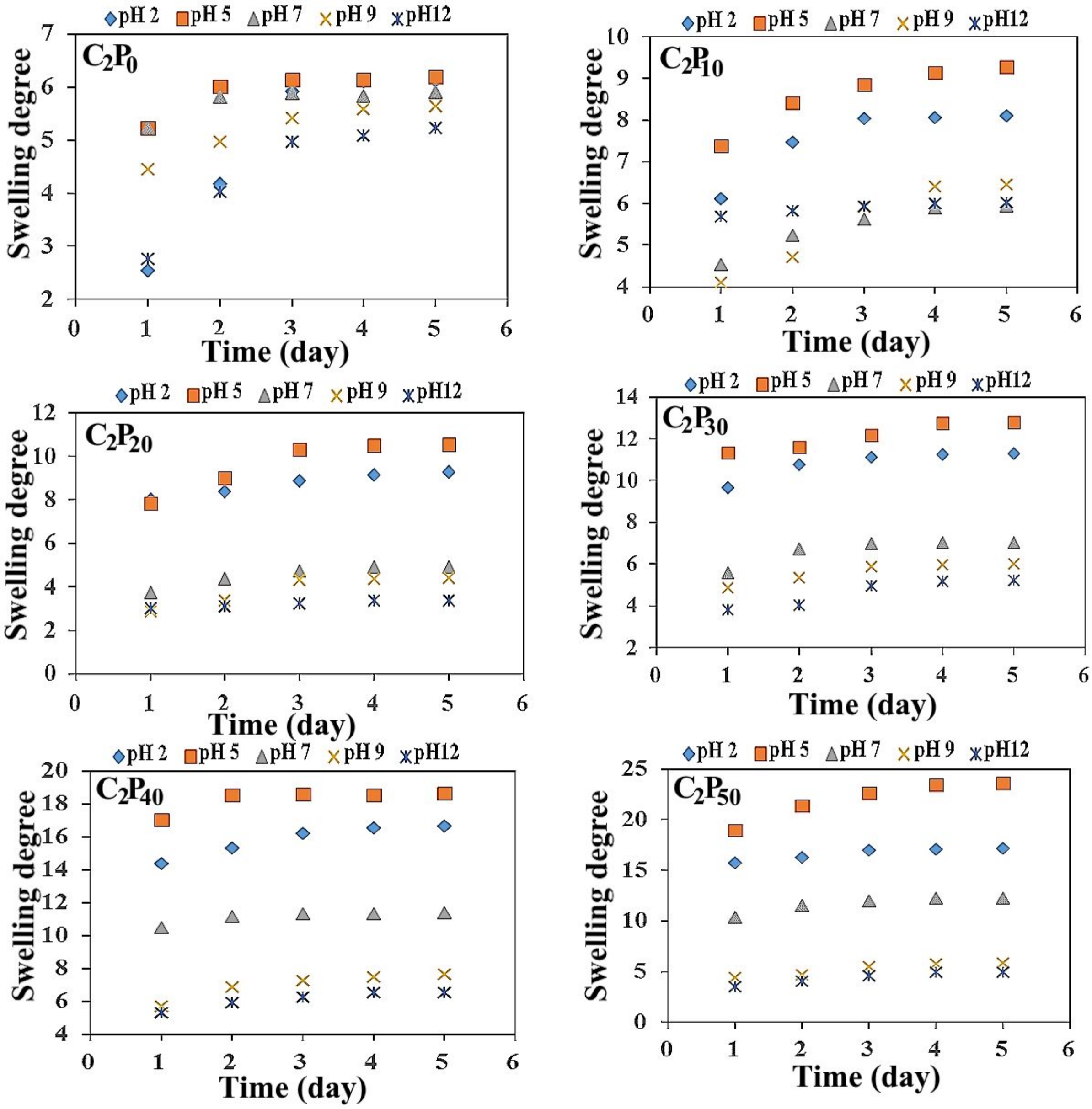

Figure S2. Swelling kinetic curves of PVA and PVA/CS hydrogels synthesized using two F–T cycles.

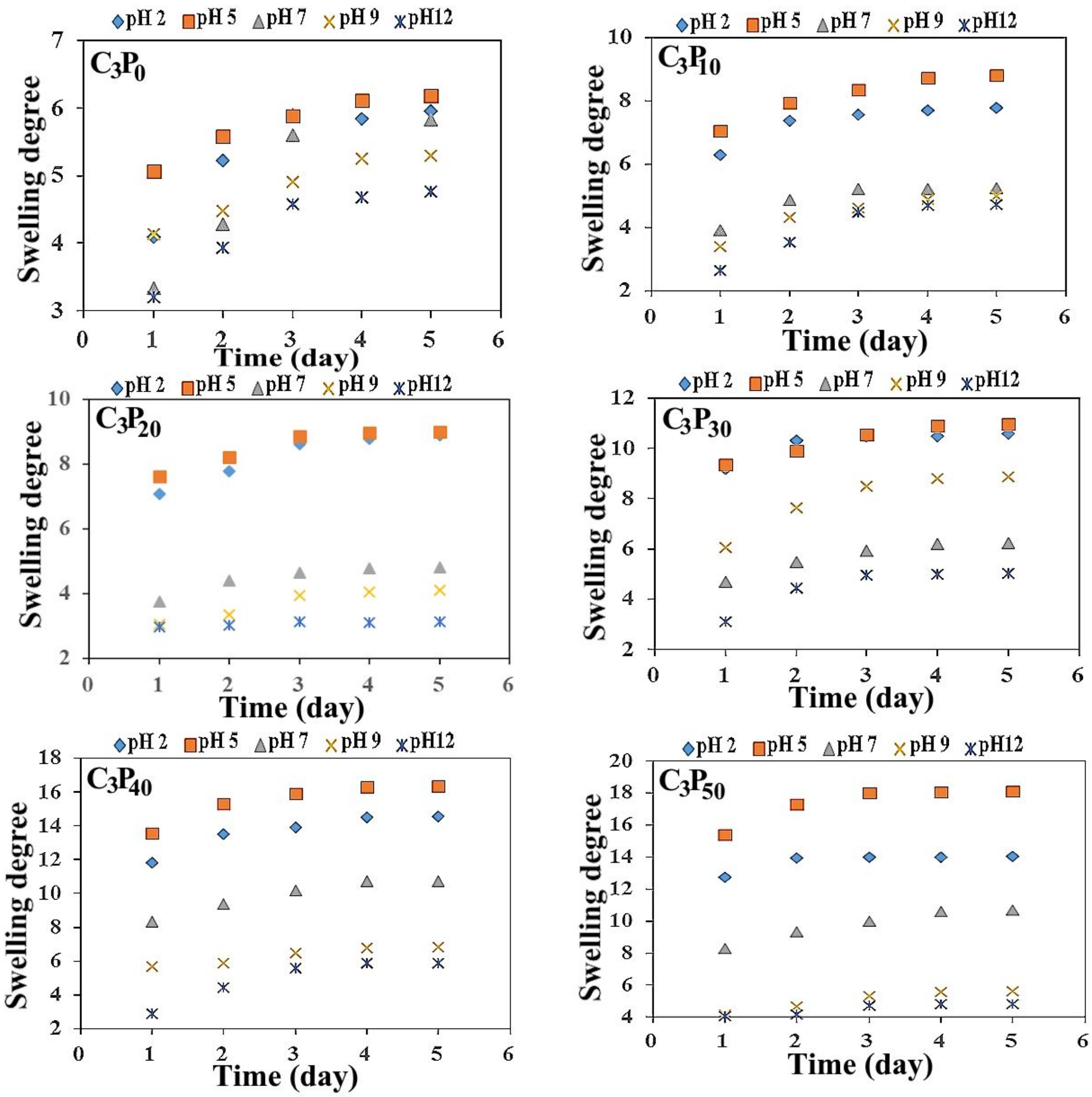

Figure S3. Swelling kinetic curves of PVA and PVA/CS hydrogels synthesized using three F–T cycles.

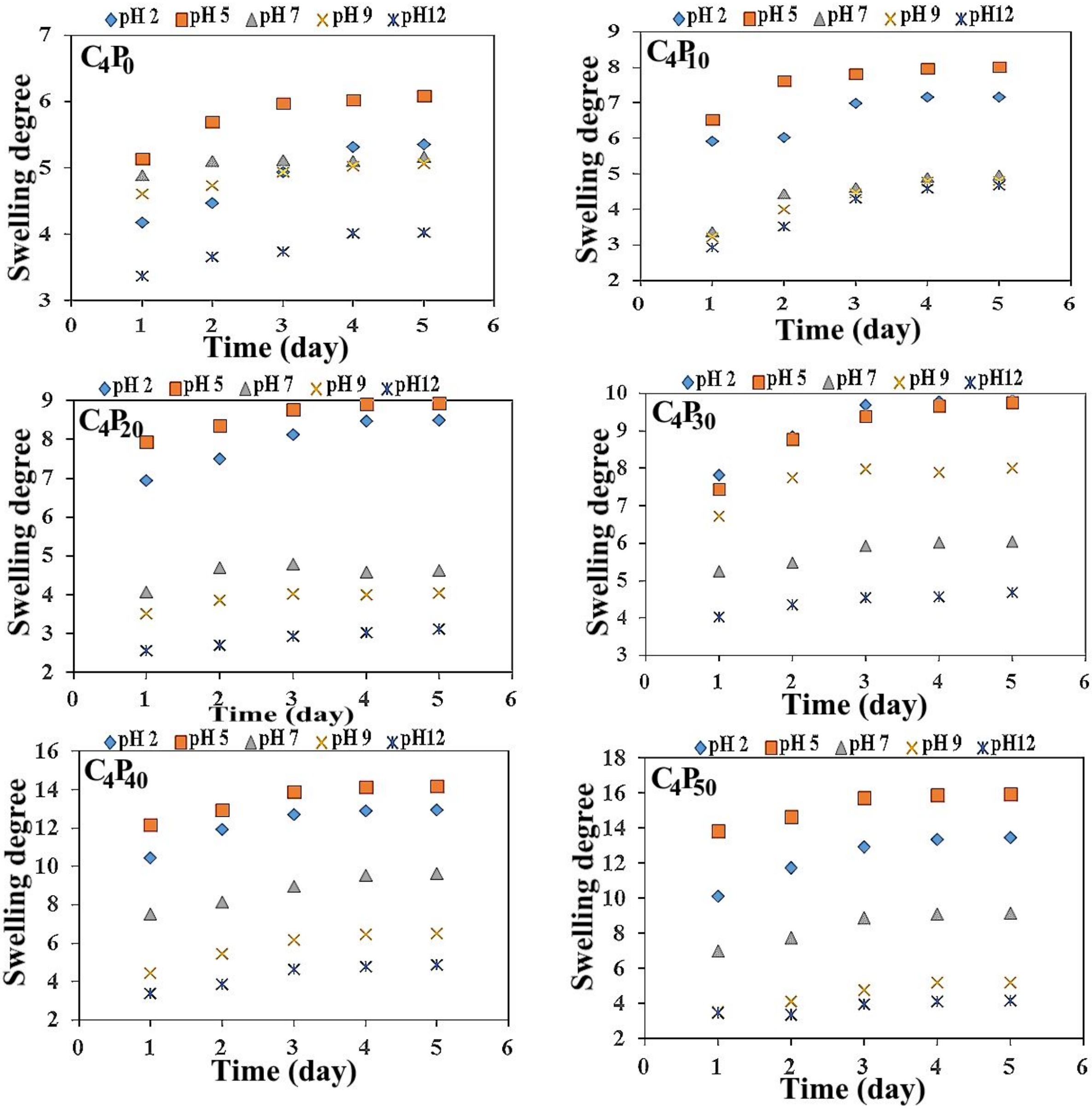

Figure S4. Swelling kinetic curves of PVA and PVA/CS hydrogels synthesized using four F–T cycles.

Table S1. Different kinetic model equations for drug release

Kinetic models	Linear equations	Plots
Zero-order	$Q_t = Q_{eq} + K_0 t$	Q_t vs t
First-order	$\ln Q_t = \ln Q_0 - K_1.t$	$-\ln Q_t$ vs t
Pseudo-second-order	$t/Q_t = 1/Ks.Q^2_{eq} + t/Q_{eq}$	t/Q_t vs t
Korsmeyer–Peppas	$\ln(Q_t/Q_{eq}) = \ln K_p + n.\ln t$	$\ln Q_t/Q_{eq}$ vs lnt

where; K_0, K_1 and K_s are swelling parameters describing zero order constant, first order constant and *Pseudo-second-order* constant, respectively. In all models, Q_t parameter relates to the swelling data anytime. In Korsmeyer–Peppas model, K_p is aconstant describing the water-sample interaction and n is the release exponent describing the transport mechanism and Q_t/Q_{eq} is the fraction of swelling data anytime.[S1-S3]

Table S2. Correlation coefficient (R^2) values of the swelling kinetics of hydrogels according to different models.

Sample	Zero-order release model	First-order release model	Pseudo-Second Order	Korsmeyer–Peppas model	ANN Model
$C_2P_{20}pH2$	0.553	0.596	0.964	0.953	0.987
$C_2P_{20}pH5$	0.516	0.605	0.968	0.955	
$C_2P_{20}pH7$	0.577	0.627	0.973	0.967	
$C_2P_{20}pH9$	0.584	0.643	0.985	0.962	
$C_2P_{20}pH12$	0.615	0.652	0.987	0.965	
$C_3P_{40}pH2$	0.104	0.448	0.938	0.940	
$C_3P_{40}pH5$	0.118	0.484	0.942	0.944	
$C_3P_{40}pH7$	0.134	0.516	0.953	0.949	
$C_3P_{40}pH9$	0.147	0.552	0.967	0.953	
$C_3P_{40}pH12$	0.166	0.567	0.965	0.957	

References:

(S1) Ghauri, Z. H.; Islam, A.; Qadir, M. A.; Gull, N.; Haider, B.; Khan, R. U.; Riaz, T. Development and Evaluation of pH-Sensitive Biodegradable Ternary Blended Hydrogel Films (Chitosan/Guar Gum/PVP) for Drug Delivery Application. *Sci. Rep.* 2021, *11*, 21255.

(S2) Ilgin, P.; Ozay, H.; Ozay, O. A New Dual Stimuli Responsive Hydrogel: Modeling Approaches for the Prediction of Drug Loading and Release Profile. Eur. Polym. J. 2019, 113, 244–253.

(S3) Erikci, S.; van den Bergh, N.; Boehm, H. Kinetic and Mechanistic Release Studies on Hyaluronan Hydrogels for Their Potential Use as a pH-Responsive Drug Delivery Device. Gels 2024, 10, 731.
